# Structure of the magma plumbing system beneath Semisopochnoi Island (Aleutian Arc) inferred from seismic tomography

**DOI:** 10.1038/s41598-022-14794-7

**Published:** 2022-06-24

**Authors:** Galina Yaroshenko, Ivan Koulakov, Nassir Al-Arifi, Saleh Qaysi, Sami El Khrepy

**Affiliations:** 1 Trofimuk Institute of Petroleum Geology and Geophysics SB RAS, Prospekt Koptyuga, 3, Novosibirsk, Russia 630090; 2 Novosibirsk State University, Pirogova 2, Novosibirsk, Russia; 3 Institute of the Earth’s Crust SB RAS, Irkutsk, Russia; 4 Chair of Natural Hazards and Mineral Resources, Geology and Geophysics Department, King Saud University, P.O. Box 2455, Riyadh, 11451 Saudi Arabia; 5 Seismology Department, National Research Institute of Astronomy and Geophysics, NRIAG, Helwan, 11421 Egypt

**Keywords:** Geology, Geophysics, Seismology, Volcanology

## Abstract

Semisopochnoi Island is a remote and little-studied volcanic island in the western part of the Aleutian Arc. The existence of several active volcanic centers and a 5000–7000-year-old large caldera makes this island an important site for volcanic hazard assessment in the Northern Pacific. Based on local seismicity data recorded by six permanent seismic stations, we created the seismic tomography model, including the 3D distributions of *Vp*, *Vs*, and *Vp*/*Vs* ratios to a depth of 10 km. This model provides the first geophysical insight into the interior structure of Semisopochnoi Island and sheds light on the processes in the magma plumbing system beneath all volcanic centers on the island. At depths of 5–10 km, we observed a columnar-shaped high *Vp*/*Vs*-ratio anomaly below the caldera in the central part of the island, which likely represents the steady magma conduit. This conduit is headed by a prominent high *Vp*/*Vs*-ratio anomaly located 3–5 km directly below the caldera, which represents the magma reservoir feeding Cerberus and other Holocene-aged volcanic centers on Semisopochnoi Island.

## Introduction

**Figure 1:**
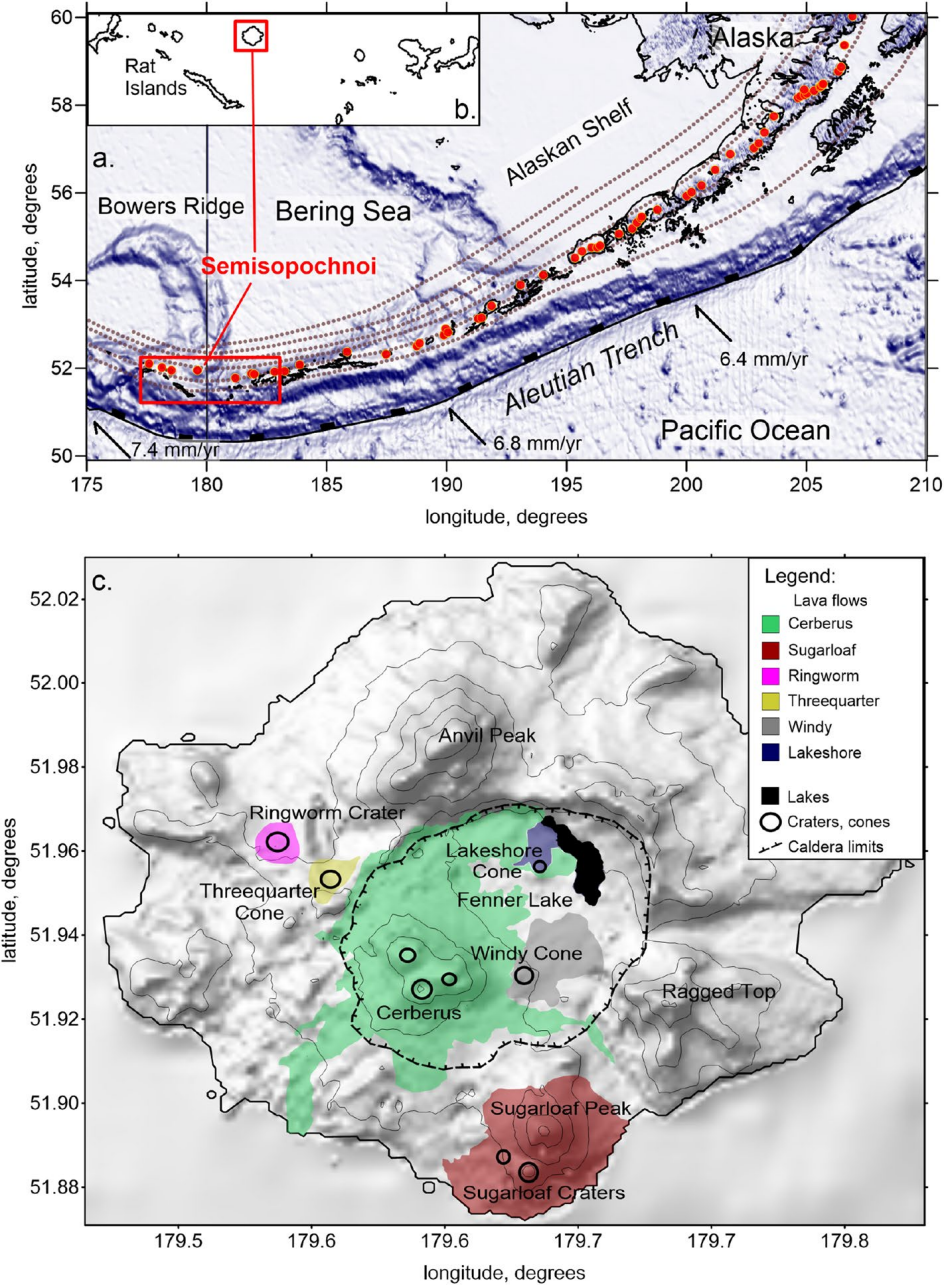
Location and general structural units of Semisopochnoi Island. (**a**) Bathymetry map of the Aleutian Arc with the location of the main Holocene-aged volcanoes (red dots), the shape of the subducted Pacific Plate (brown dotted lines), and plate movement vectors^16^. (**b**) Location of Semisopochnoi in the Rat Island Group. (**c**) Topography map of Semisopochnoi Island indicating lava flows and the main structural elements (modified from Coombs et al.^12^). The figure was generated using the software Surfer (version 13, http://www.goldensofware.com/products/surfer).

The high level of volcanic and seismic activity of the Aleutian Arc (Fig. 1a) may possibly pose serious problems for the entire North Pacific region^1,2^. The volcanic islands of the Aleutian Arc are characterized by the presence of traces of violent explosive eruptions in the recent geological past^3^. Potential eruptions and earthquakes in this area can pose a threat to people living on the islands and dense aviation routes^4^. To respond to this threat in a timely manner, networks of telemetric seismic stations operate permanently in most key Alaskan and Aleutian locations and continuously transmit data in real time to the office of the Alaska Volcano Observatory (AVO)^5^. These stations have been in operation for decades, so they provide useful information on the internal processes and structures of active volcanoes. Data from these networks have been used to create seismic tomography models for several Aleutian and Alaskan volcanoes, which has helped better understand the evolution of the magma sources^6–11^.

Besides natural hazard issues, geophysical studies of structures beneath volcanic areas are important for the exploration of geothermal resources, which are some of the most prospective sources of renewable carbon-free energy. Although most of the Aleutian Islands are inhabited, geothermal power plants built on them might also be used to generate hydrogen, which is in increasing demand due to the ecological challenges facing the world.

Here, we consider the remote and little-studied Semisopochnoi Island located in the western part of the Aleutian Arc. Episodic volcanic activity involving multiple vents makes this island an important site for the volcanic hazard assessment of the Western Aleutians. Furthermore, the existence of a large 5000–7000-year-old caldera^12^ indicates that the volcanoes on Semisopochnoi Island could potentially produce catastrophic explosive eruptions with regional and, possibly, global impacts. Due to the difficulty of access, a limited number of experimental studies have been performed there. The first geological map of Semisopochnoi Island was compiled by Robert Coats and his team based on two expeditions conducted in the 1940s and the 1950s^13^. Later geochemical studies by De Long^14^ in 1974 and De Long et al.^15^ in 1985 were mostly based on the analyses of rock samples collected by Coats’ expeditions. A subsequent large field campaign was conducted in 2005 by Michelle L. Coombs’ team, who considerably enhanced the geological map of the island and acquired a large rock collection for petrological, geochemical, and geochronological studies. Coombs et al.’s^12^ report, which summarizes the results of these studies, is the main source of information for the short geological overview presented in the next section.

Most importantly for us is the permanent network of telemetric seismic stations that the Coombs’ team deployed during the 2005 campaign, which has operated continuously since then. Using the arrival times of P- and S-waves from the local seismicity recorded by this network, we created a seismic tomography model of the crust beneath Semisopochnoi Island. As far as we know, this is the first geophysical image of the crustal structure beneath Semisopochnoi Island. The importance of this study is that it sheds light on the details of the magma plumbing systems of the Semisopochnoi Island volcanoes. Moreover, a comparison of the results of this study with those of other Aleutian volcanoes will provide a better understanding of the common principles of the Aleutian Arc’s functioning arc volcanism.

### Geological overview

Semisopochnoi Island belongs to the Rat Island Group in the western part of the Aleutian arc. Morphologically, Semisopochnoi Island is the largest subaerial volcano in the western Aleutian Arc. The 19 × 17 km island is almost circular (Fig. 1b). The island’s relief is dominated by the 6–7 km diameter circular caldera and several stratovolcano cones, which give the island its name (*semisopochnoi* means Seven Hills in Russian). As for most of the Aleutian Islands, Semisopochnoi Island volcanism is associated with the ongoing oblique subduction of the Pacific Plate at 74 mm/yr^16^. Semisopochnoi Island is located on the southern extent of the Bower Ridge, a relict subduction zone, which may determine a special character of volcanism in this area^17^.

The Pleistocene-aged basement of the island is a large predominantly basaltic shield volcano^12^. The exact age of the volcano is unknown, but it is estimated to be older than several hundred thousand years. The island’s highest point is Anvil Peak, a late-Pleistocene double-headed cone that occupies most of the northern part of the island. Two other peaks on the eastern part of the island, Ragged Top and Pochnoi, are extinct Pleistocene-aged volcanoes that are thought to have not been active during the Holocene^12^.

During the Holocene, the composition of the volcanic activity became strongly variable, ranging from basaltic to dacitic. A strong explosive eruption that occurred 5000–7000 years ago^12^ destroyed the central part of the island and formed the caldera. The ignimbrites associated with this eruption created a layer across the entire island, ranging in thickness from several meters up to dozens of meters^12^. The edge of the caldera is well expressed (dotted line in Fig. 1b), except to the southwest, where it is covered by post-caldera lava flows. The oldest lavas on the floor of the caldera are andesitic to dacitic, but they are mostly covered by younger, predominantly basaltic to basalt-andesitic composition lavas and tephras.

The post-caldera volcanic centers developed independently of the caldera geometry; they were controlled by subduction-related tectonic processes and relatively fast magma ascent through a fractured lithosphere^18^. Therefore, the most recently active vents are located both inside and outside the caldera and are apparently not associated with the caldera’s borders. Inside the caldera, the main volcanic activity is represented by the three peaks of Mount Cerberus, all with well-expressed craters. Voluminous, mostly basalt-andesitic composition lava flows from these vents cover most of the caldera surface, with some reaching the sea (green area in Fig. 1b). On the northeastern flank of Mount Cerberus, small fresh lava flows that are dozens of years old have been found. Windy and Lakeshore, monogenic cones located in the northeastern part of the caldera in the vicinity of Fenner Lake (Fig. 1b), represent two other intra-caldera vents that have produced relatively small lava flows^12^.

Outside the caldera, most of the Holocene activity, which was basaltic, occurred at Sugarloaf Peak on the southernmost point of the island^12^. Its young satellite peak on the southern flank, Sugarloaf Head, and associated cinder cones produced large basaltic lava flows that propagated to offshore areas (red area in Fig. 1b). Two smaller Holocene-aged volcanic vents, Ringworm crater and Threequarter Cone, are located in the northwestern part of the island. These monogenic cones have identical andesitic compositions and similar stratigraphic positions^12,13^.

Close to the Windy and Lakeshore Cones, a few relatively weak hydrothermal 21°–24 °C springs were found^12^, which appear to be the only hydrothermal activity on the island. The water from these springs is of meteoric origin; however, Evans et al.^19^ found traces of dissolved magmatic CO_2_.

Because of its remoteness and lack of population, historical volcanic eruptions on Semisopochnoi Island are not well documented. Some volcanic activity was mentioned in historical records dating from 1772, 1790, 1792, 1830, and 1873, but no details of these events were described. Since the 1980s, when satellite images became available, it has been possible to observe the volcanic activity more regularly. The first instrumentally observed eruption occurred on April 13, 1987. A 90-km long ash plume was detected in satellite images^20,21^. AVO reports recorded minor ash explosions in September and October 2018; between July 16 and August 24, 2019; from December 2019 through to mid-March 2020; and from February to June 2021.

The background seismicity beneath Semisopochnoi Island is relatively weak^5,22^. However, strong seismic unrest took place beneath Semisopochnoi Island in June 2014^23^. Several thousand recorded volcano-tectonic events may have marked magmatic intrusion; however, none resulted in a magmatic eruption. Interferometric synthetic aperture radar (InSAR) observations were used to evaluate ground deformations during this unrest, and DeGrandpre et al.^24^ reported more than 25 cm of total inflation during 2003 and up to June 26, 2014, of which more than half occurred in the ten days between June 15 and 26, 2014. In June, the inflation rate reached 6 mm per day^24^, with inflation occurring as a simple radial pattern centered inside the caldera with a slight northeast–southwest oblique skew. After June 26, 2014, the ground deformation almost ceased until resuming during another episode of seismic activity from January to May 2015^23,24^. Although the total number of events was considerably lower compared to 2014, some of the events had relatively large magnitudes between *M* 2.0 and 2.8, with depths of up to 10 km^22^. Based on the observed InSAR measurements and relocated seismicity, DeGrandpre et al.’s^24^ inverse modeling estimated the locations of the deformation sources for both episodes. They associated the observed ground deformations with magma influx from a depth of ~ 8 km directly below the caldera. Until now, these results, together with the seismicity distribution, were the only geophysical data that provided information on the structure beneath Semisopochnoi Island.

### Data

**Figure 2:**
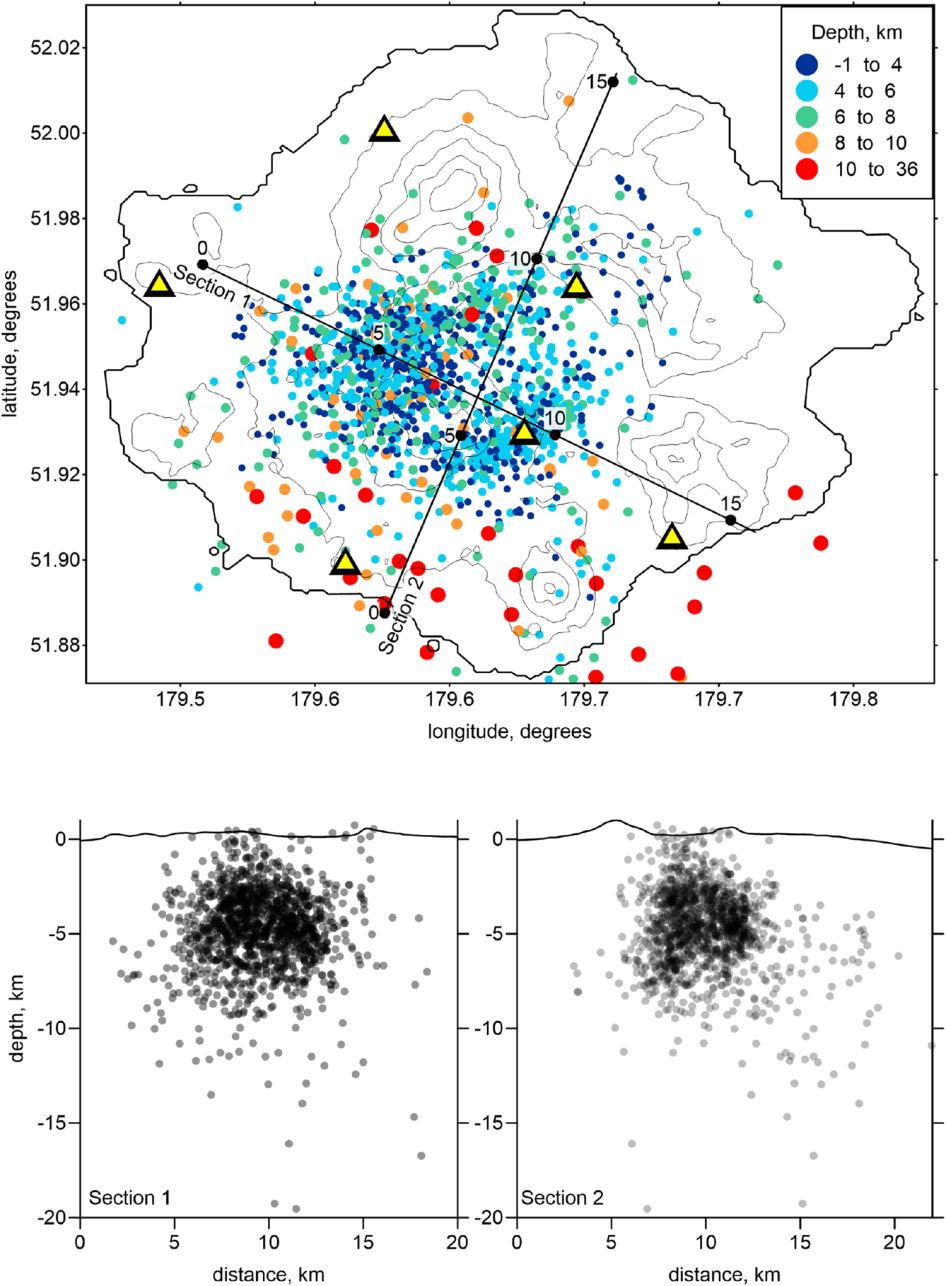
Distribution of the seismicity used in this study shown in map view and in two vertical sections. The locations of the events correspond to the final solutions after the tomographic inversion. The seismic stations used in this study are indicated by the yellow triangles. The figure was generated using the software Surfer (version 13, http://www.goldensofware.com/products/surfer).

In this study, we used the local seismicity *P*- and *S*-wave arrival times from January 1, 2013, to December 31, 2017, provided by the AVO. These data were recorded by six permanent Semisopochnoi Island stations, installed in September 2005^22^. The catalog contained information on 3410 events with 11,711 *P*- and 9803 *S*-wave arrival times (average of 6.3 picks per event). We used several criteria to select the data for the tomography, including (1) the distance from an event to the nearest station should be less than 20 km, (2) the total number of *P*- and *S*-wave picks per event should be equal to or larger than 4, and (3) the time residual after locating the events in the starting 1D velocity model should be smaller than 0.5 s. After applying these criteria, the number of data was significantly reduced to 1784 events (6142 *P*- and 5606 *S*-wave picks; 6.6 picks per event). The locations of the events after running the full tomography procedure are shown in map view and two vertical sections in Fig. 2. Note that some of events in the vertical sections appear to be above the surface, because we projected the events onto the profile plane from a certain band, where higher topography features than those on the profile may exist.

The low number of stations used in the current study compared to most other tomography studies may have resulted in the poor source locations and the low quality of the tomographic images. However, other studies with similar station configurations have provided fair-quality results, such as eight stations on Avacha volcano^25^, eight stations on Nevado del Huila^26^, and four stations on Udina^27^. All of these studies underwent subsequent extensive testing that proved the reliability of the models. Following the same strategy, we undertook a number of tests to demonstrate the resolution limitations of our case.

The distributions of the P and S wave velocities, as well as the *Vp*/*Vs* ratio, were calculated using the LOTOS code for passive-source tomography^28^, which was previously implemented in a number of volcanoes with similar data configurations. Details on the algorithm are presented in “Methods” section.

## Results

The results of the synthetic tests allowed us to assess the spatial resolution of the model and the accuracy of the source locations. This was particularly important given the relatively low number of stations. For the synthetic modeling, we reproduced all the conditions of the experimental data processing, including the same calculation steps and controlling parameters. Here, we present two groups of tests aimed at separately assessing the vertical and horizontal resolutions. Synthetic travel times using the same source–receiver pairs used for the experimental dataset were calculated in a predefined synthetic model using bending ray-tracing. These travel times were perturbed by random noise, with an average deviation of 0.05 and 0.1 s for the *P*- and *S*- wave data, respectively. This provided a variance reduction similar to the inversion of the experimental data. We then “forgot” all the information about the true velocity model, source coordinates, and origin times, and reproduced all the experimental data processing steps, including finding the initial locations of the sources in the starting 1D model.

**Figure 3:**
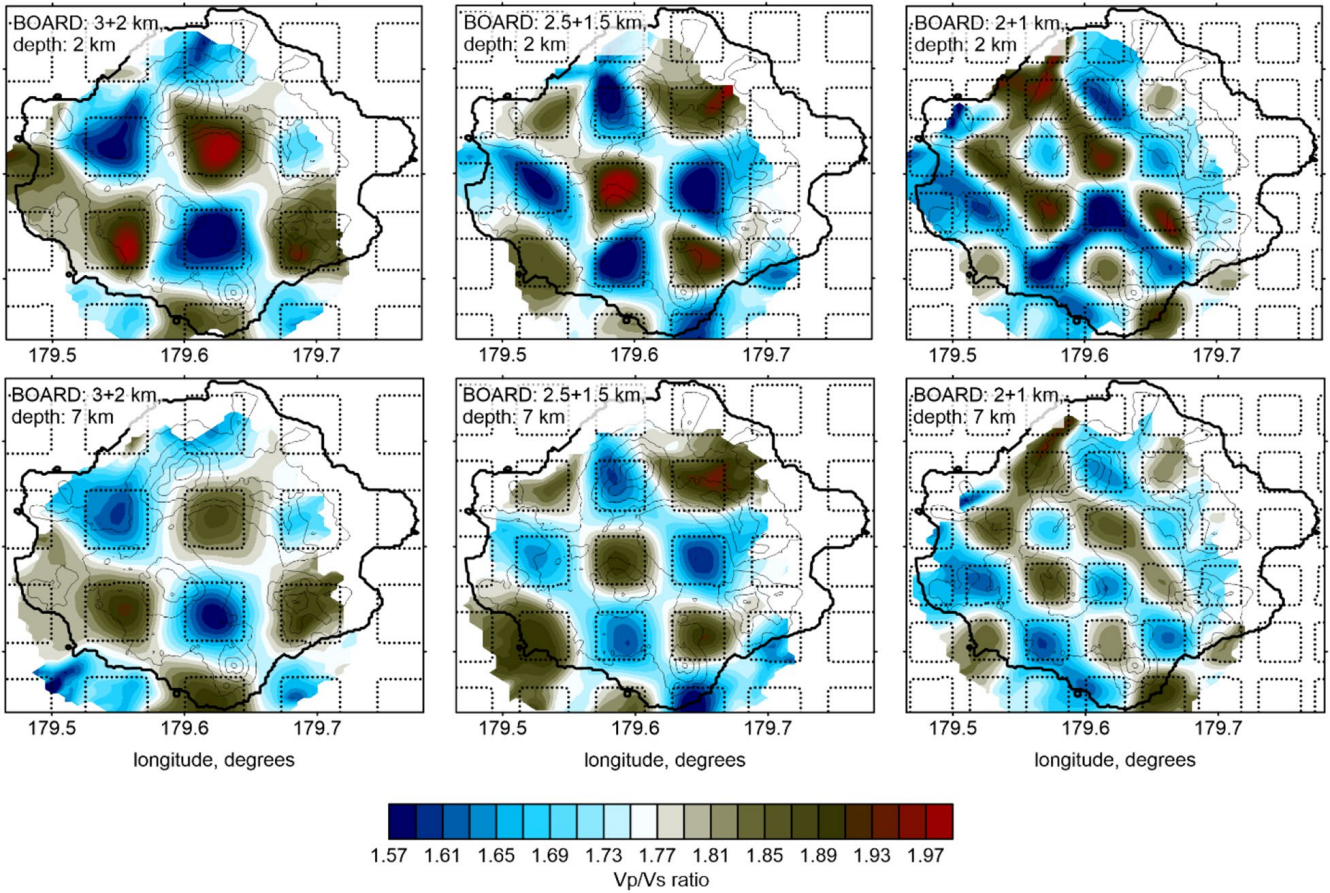
Resulting distributions of the *Vp*/*Vs* ratio after the recovery of three horizontal checkerboard tests. The sizes of the synthetic anomalies and intervals are indicated in the left corner of each map. The locations of the synthetic anomalies are indicated by the dotted lines. The topography is contoured at 200 m intervals. The figure was generated using the software Surfer (version 13, http://www.goldensofware.com/products/surfer).

The results of three horizontal checkerboard tests with different sized anomalies are shown in Fig. 3. In all cases, the anomalies were defined as unlimited columns, and their size and the spacing between them are indicated in the figure captions. The anomaly amplitude was ± 7%, and it had the opposite signs for d*Vp* and d*Vs*. The recovered values of the *Vp*/*Vs* ratio are shown in two horizontal sections at depths of 2 and 7 km. The 3-km and 2.5-km anomalies were robustly resolved at both depth levels, whereas the 2-km anomalies appeared to be slightly smeared. This test indicated the minimum size of the anomalies that could be resolved in different parts of the study area, based on the existing data.

**Figure 4:**
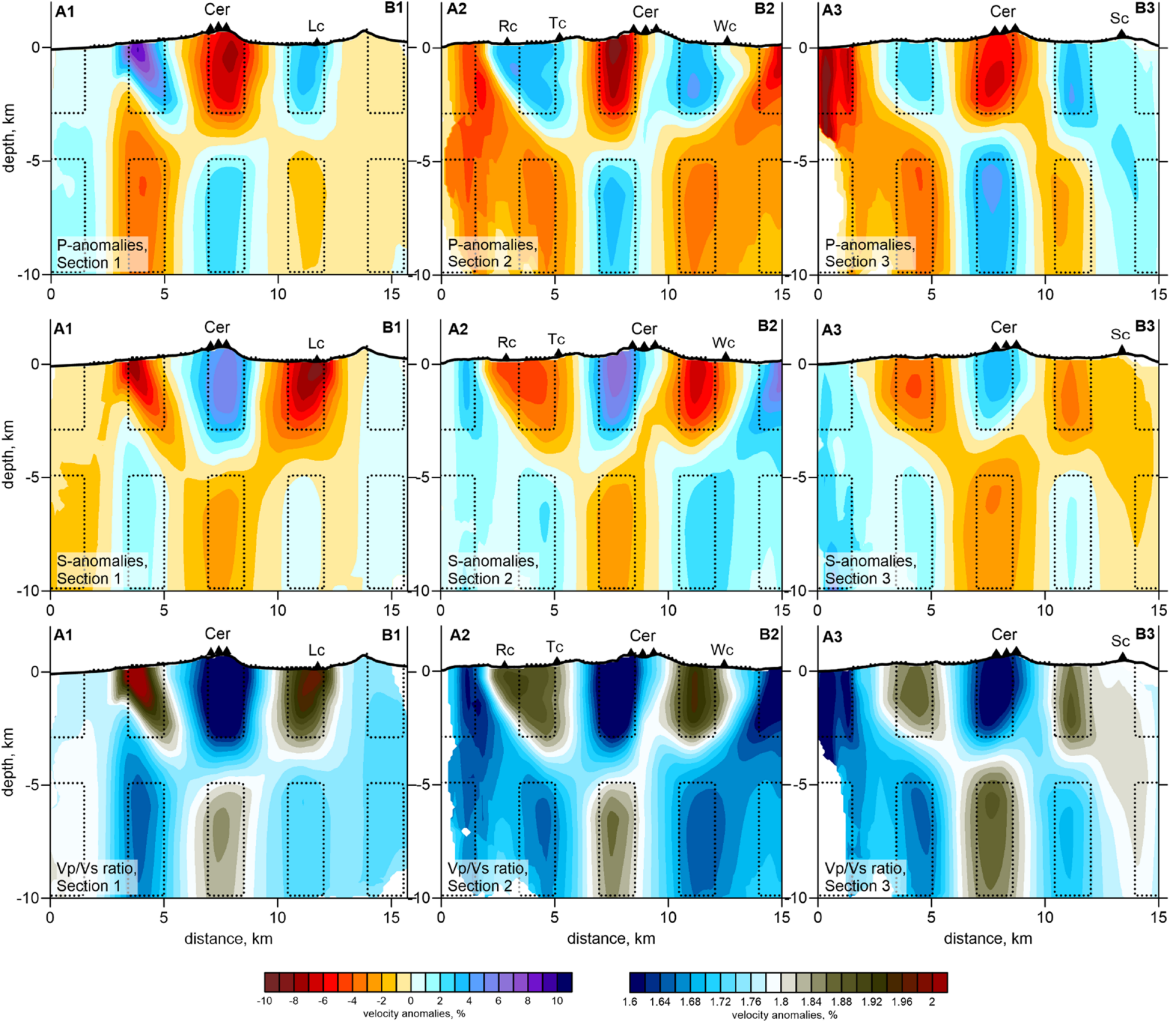
Resulting distributions of d*Vp*, d*Vs*, and *Vp*/*Vs* ratios after the recovery of three synthetic models defined along three vertical sections (the same as those used to present the main results in Figs. 6 and 7). The synthetic anomalies are indicated by the dashed lines. The projections of the main volcanic vents are shown as black triangles. *Cer* cerberus stratovolcano, *RC* ringworm cone, *Tc* threequarter cone, *Wc* windy cone, *Lc* lakeshore cone, *Sc* sugarloaf cones. The figure was generated using the software Surfer (version 13, http://www.goldensofware.com/products/surfer).

In another test, three synthetic models were defined along three vertical sections, the same as those used to present the main results in Fig. 4. In this case, the models consisted of two layers of alternating anomalies with the sign change interval at a depth of 3–5 km. In the direction along the section, the size of the anomalies was 1.5 km, and the empty spacing between the anomalies was 2 km. The thickness of the anomalies in the direction across the section was 6 km. Similar to the previous case, the ± 7% amplitude anomalies of the *P*- and *S*-wave velocities had opposite signs, which enabled contrasted variations of the *Vp*/*Vs* ratio. All these models were robustly recovered in the central part of the model, including the 3–5 km deep transition. However, instead of narrow anomalies separated by large interval of zero values, we observe some smearing, when the transitions between anomalies appear to be smooth. Similar effect might exist in the case of the experimental data inversion.

**Figure 5:**
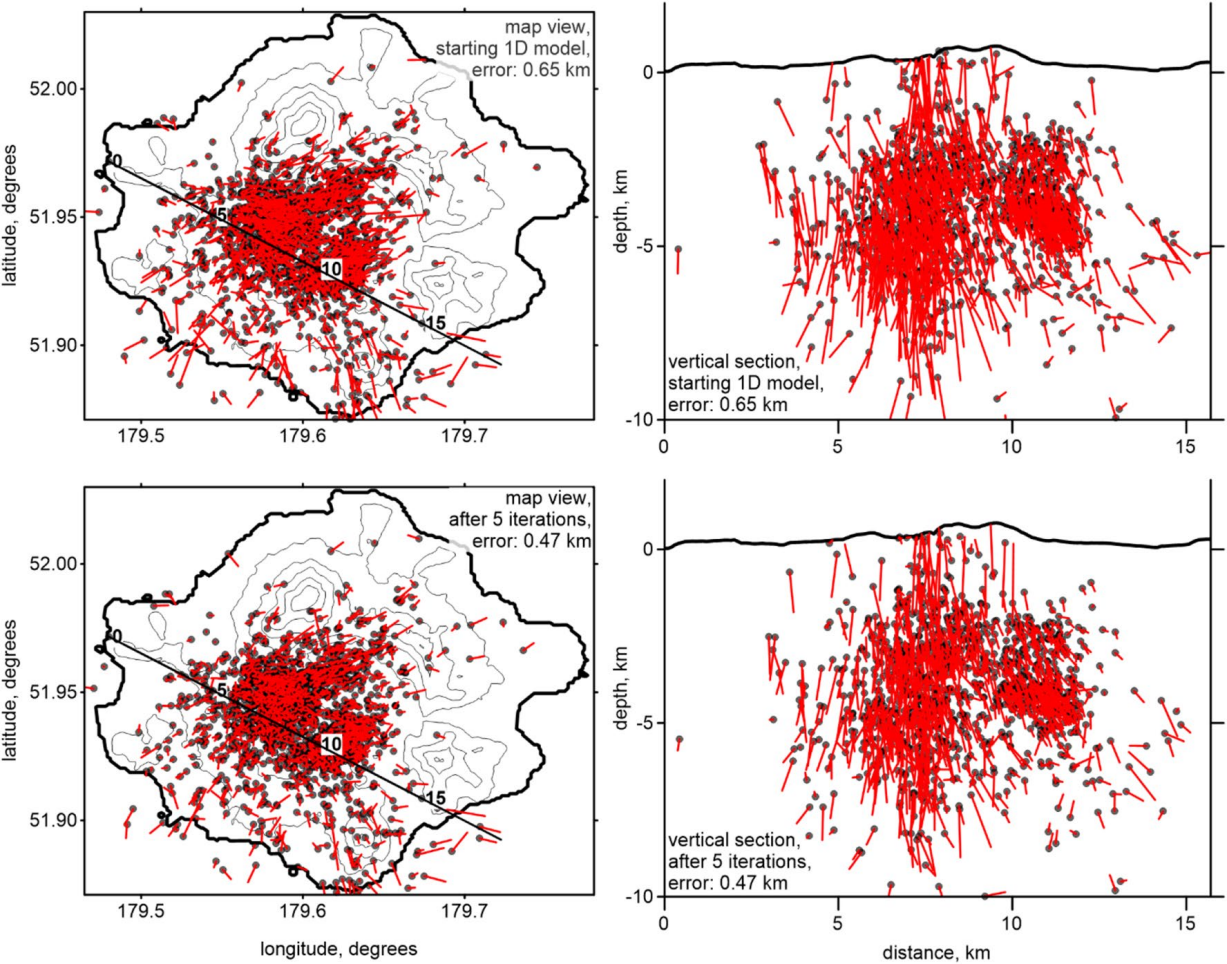
Mislocations of the sources during the synthetic modeling (for the model indicated in the right-hand column in Fig. 4) shown in map view and a vertical section. The upper row shows the location results using the start of the 1D model, and the lower row shows the location results of the final 3D velocity model. The black dots indicate the location of current events, and the red bars indicate the true locations. The mean errors of the source locations are indicated in the figure captions. The figure was generated using the software Surfer (version 13, http://www.goldensofware.com/products/surfer).

The synthetic tests provided the opportunity to estimate the accuracy of the source locations. During the recovery procedure, the true source locations were presumed to be unknown; therefore, the difference between the final and true locations provided an adequate image of the real uncertainty of the source locations during the experimental data inversion. An example of source mislocations for the vertical checkerboard along Sect. 3 (shown in the right-hand column of Fig. 4) is presented in Fig. 5. After locations in the starting 1D model, the mean error was 0.65 km; however, after five iterations of the simultaneous inversions of the source and velocity parameters, the error was reduced to 0.47 km. As expected, the uncertainty of the source depth locations was larger than that of the horizontal coordinates. Note that despite such significant errors and outliers, the velocity model was correctly recovered.

**Figure 6:**
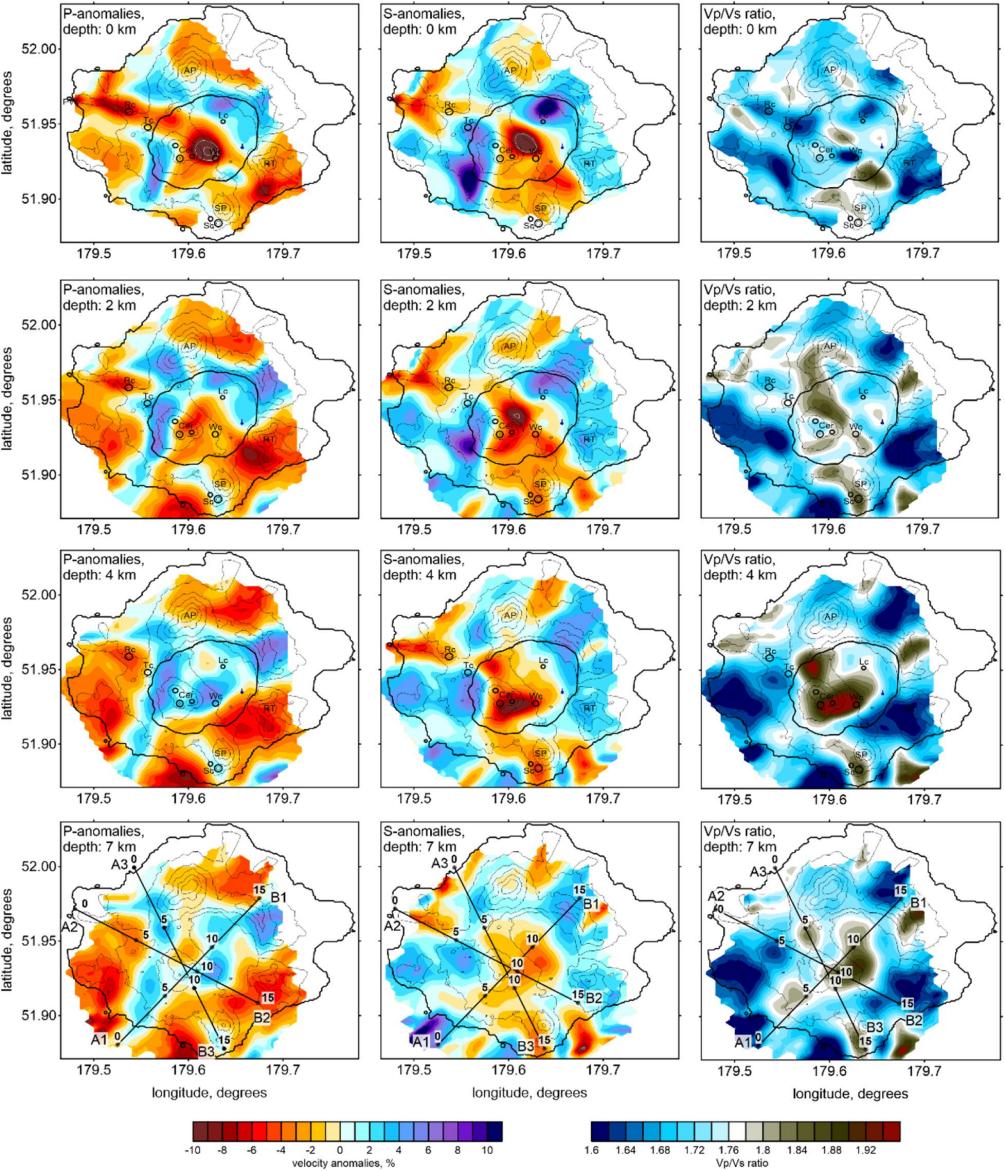
Distribution of the *Vp* and *Vs* anomalies and the *Vp*/*Vs* ratios obtained from the inversion of the experimental data in four horizontal sections. The locations of the vertical sections are indicated in the maps at a depth of 7 km. The topography is contoured at 200 m intervals. *Cer* cerberus stratovolcano, *RC* ringworm cone, *Tc* threequarter cone, *Wc* windy cone, *Lc* lakeshore cone, *Sc* sugarloaf cones, *SP* sugarloaf peak, *AP* anvil peak, *RT* ragged top. The figure was generated using the software Surfer (version 13, http://www.goldensofware.com/products/surfer).

**Figure 7:**
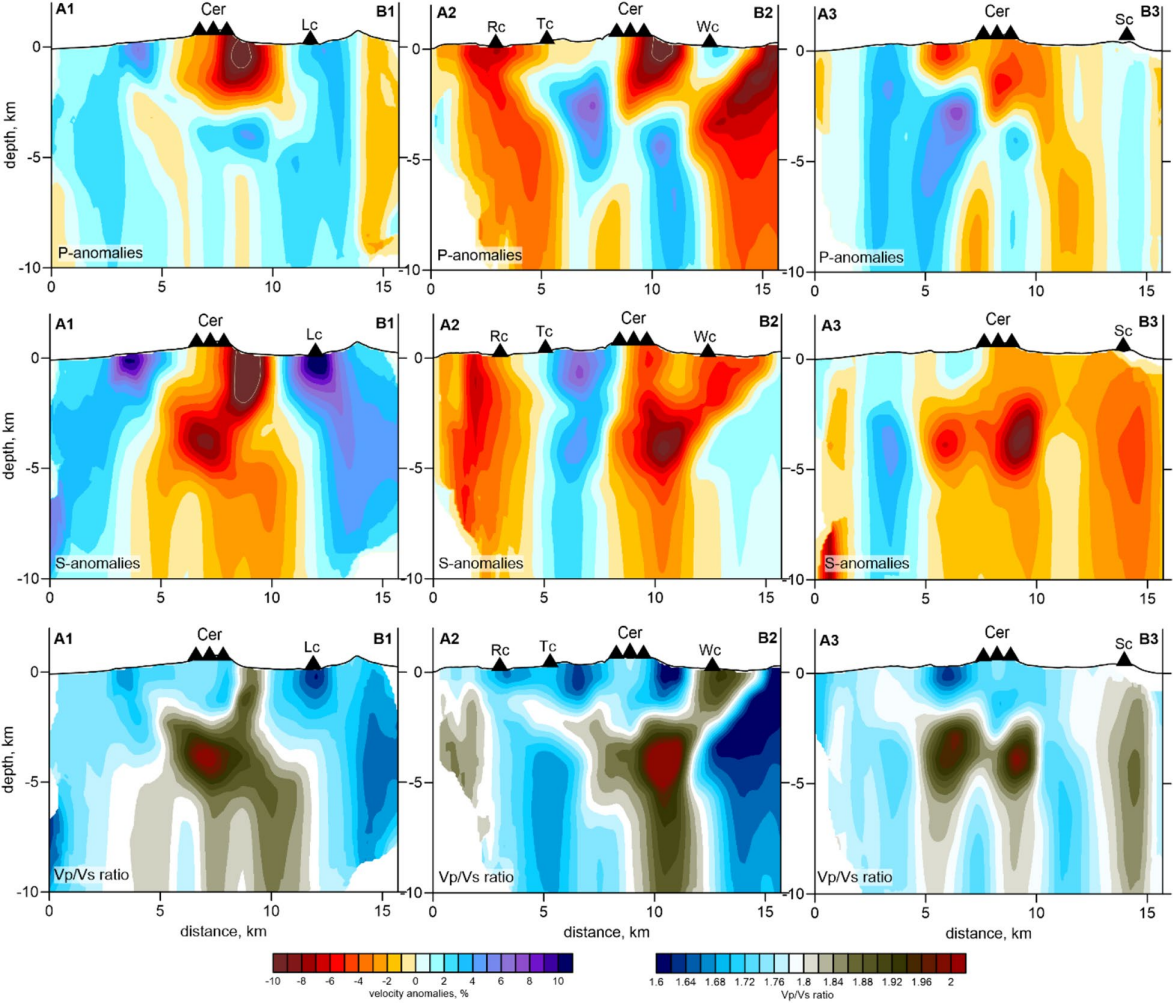
Distribution of the *Vp* and *Vs* anomalies and the *Vp*/*Vs* ratios obtained from the inversion of the experimental data in three vertical sections, as indicated in Fig. 6. The projections of the main volcanic vents are shown as black triangles. *Cer* cerberus stratovolcano, *RC* ringworm cone, *Tc* threequarter cone, *Wc* windy cone, *Lc* lakeshore cone, *Sc* sugarloaf cone. The figure was generated using the software Surfer (version 13, http://www.goldensofware.com/products/surfer).

The results of the experimental data inversion are presented in Figs. 6 and 7 in four horizontal and three vertical sections with the *Vp* and *Vs* anomalies and the *Vp*/*Vs* ratios, which were calculated by the division of the resulting absolute *P*- and *S*-wave velocities. The stability of this method for calculating *Vp*/*Vs* was confirmed by the series of synthetic tests described above. The final locations of the sources of this velocity model are shown in map view and vertical sections in Fig. 2.

## Discussion

The derived d*Vp*, d*Vs*, and, especially, *Vp*/*Vs*-ratio anomalies provided important information about the magma plumbing processes beneath the Semisopochnoi Island volcanoes. Based on experimental studies^29^ and several previous tomographic studies of other volcanoes^8–11,25,30,31^, some common relationships between seismic parameters and rock properties can be proposed. For example, *P*-wave velocity is mostly sensitive to rock composition, whereas *S*-wave velocity is mostly affected by the presence of a liquid phase (e.g., melt and volatiles). Therefore, semi-molten magma stores and conduits are usually associated with anomalies with higher d*Vp*, lower d*Vs*, and a very high *Vp*/*Vs* ratio. For the most active volcanoes worldwide, where *P*- and *S*-wave velocities are available, such relationships are very helpful for imaging magma reservoirs and conduits^8–11,25,30,31^^.^

However, this pattern might not be valid at shallow depths, as there are too many different processes affecting the mechanical properties of the rocks, such as the interlayering of soft sediments and rigid lava flows, the penetration of meteoric water, and hydrothermal alteration. In this case, a separate interpretation of *Vp* and *Vs* appears to be more informative than consideration of the *Vp*/*Vs* ratio. Indeed, as shown in Fig. 6 in the sections at 0 and 2 km deep, most of the Holocene craters are located inside or on the border of the low-velocity *Vp* and *Vs* anomalies, but they are almost not expressed by the *Vp*/*Vs* ratios. A prominent low *Vp* and *Vs* anomaly is located in the center of the caldera, directly below Windy Cone. This anomaly may represent the accumulation area of the soft volcaniclastic rocks that filled the caldera and/or a hydrothermally altered zone. The Cerberus Cones are located on the border between the low-velocity anomaly and a high-velocity pattern, representing rigid, highly consolidated rock. Such a mechanically differential contact between media represents a weak zone, through which magma ascent is most plausible. An association of active volcanic vents with borders between high- and low-velocity zones is observed in many volcanoes (e.g., La Palma volcano^32^ and Tolbachik volcano^33^).

In the deeper sections at 4 and 7 km (Fig. 6), the general configuration of the anomalies appears considerably different compared to the shallow sections. Here, the *Vp* and *Vs* anomalies generally look inversely correlated. The positive *Vp* anomaly inside the caldera coexists with the strong negative *Vs* anomaly, which causes a very high *Vp*/*Vs* ratio, exceeding 2. These patterns are typical attributes of magma storage areas observed below many active volcanoes^25,30,31^. In all the vertical sections shown in Fig. 7, this anomaly is located between 3 and 5 km deep. Below this, there is a vertical anomaly with a high *Vp*/*Vs* ratio, which may represent a magma conduit connected to the upper crustal reservoir. The movement of magma through this conduit was probably the cause of the 2014 seismic unrest and the corresponding ground deformations detected by the InSAR observations^24^. This anomaly matches well with the results of the mechanical inverse modeling, indicating that the source of the magma that created the ~ 25-cm inflation was located at a depth of 5–10 km below the central part of the caldera^24^, exactly where we located the high *Vp*/*Vs*-ratio anomaly.

The vertical sections in Fig. 7 show how the Holocene-aged Semisopochnoi Island vents were fed. In “2” section of the *Vp*/*Vs* ratio, the main 3–5 km deep reservoir has several inclined upgoing branches. The left branch ascends to the Ringworm and Threequarter monogenic cones, and the right branch ascends toward the Windy Cone. However, the elevated *Vp*/*Vs*-ratio anomaly was less prominent than the periphery branches below the Cerberus stratovolcano, where most of the historical activity was observed. An explanation for this could be that most of the Cerberus eruptions were mainly basaltic. Low-viscosity basaltic magma can propagate through narrow fractures; it does not require large conduits. Therefore, the magma migration paths beneath Cerberus cannot be resolved in our model. Another explanation could be the different sensitivities of the *P*- and *S*-wave velocities at shallow depths. They are both negative below Cerberus, but the existence of other near-surface processes prevents the origin of high *Vp*/*Vs* patterns.

Another important feature is observed beneath the Sugarloaf volcanic complex, another historically active volcanic center. In all the horizontal sections below the Sugarloaf Cones, a local high *Vp* anomaly coexists with a strong low *Vs* anomaly and a high *Vp*/*Vs* ratio, a clear attribute of semi-molten magma. As shown in vertical “3” section in Fig. 7, the vertical high *Vp*/*Vs*-ratio anomaly beneath the Sugarloaf Cones appears to be isolated from the shallow magma source beneath Cerberus. It is possible that they have a common source at a greater depth that cannot be resolved with the existing data.

The structure obtained for Semisopochnoi Island in this study is consistent with a number of other tomography models derived from other Aleutian and Alaskan volcanic islands. For example, Koulakov et al.’s^11^ modeling of the structure beneath Akutan Volcano indicated a single deep conduit up to 5 km below the surface that branches at shallower depths to form a number of local conduits connected to volcanic vents and centers of geothermal activity. Koulakov et al.’s^9,31^ tomography modeling beneath Mount Spurr indicated a columnar high *Vp*/*Vs*-ratio anomaly from 4 to 5 km below the surface, which, at shallower depths, is replaced by a zone of low *Vp*/*Vs*. The existence of well-established single conduits in these cases might be explained by the steady state of the Aleutian subduction, which has remained in the same location for hundreds thousand or even millions of years. This would determine a common scenario for the development of arc volcanism that started with the long-term accumulation of basaltic material from a single conduit over millions of years. This finally produced a large shield volcano, creating a circular island. As the crust thickened, the composition of the magma became more silicic. At some point, a strong explosive eruption formed a large caldera. As the upper crust became more heterogeneous, several magma pathways were created that connected the deep conduit to the surface. As a result, volcanic eruptions of variable compositions occurred through multiple vents distributed across a large area, both inside and outside the caldera.

## Conclusion

We have presented the first seismic tomography model for Semisopochnoi Island. It provides insight into the structure beneath the island and reveals details of the magma plumbing system beneath the island’s Holocene-aged volcanoes, some of which are currently active. Because we used data from only six seismic stations, we performed a number of synthetic tests to prove the sufficient resolution of the tomography model.

In the deep part of the model, a columnar anomaly of high *Vp*, low *Vs*, and high *Vp*/*Vs* ratio revealed the location of the magma conduit, which was consistent with the location of the magma source estimated from the modeling of ground deformation during the 2014 seismic swarm^24^. Above this conduit, we observed a very high *Vp*/*Vs*-ratio anomaly reaching depths of 3–5 km located beneath the presently active Cerberus volcano, which likely represents its magma reservoir. This anomaly appears to be connected to other Holocene-aged volcanic centers located inside and outside the caldera. However, another active volcanic complex, Sugarloaf, seems to be isolated from the Cerberus magma reservoir and located above another vertical conduit.

This tomography model has potential for the exploration of geothermal energy sources on Semisopochnoi Island. The upper limit of the magma reservoir is located ~ 3 km beneath the central part of the caldera, so a 2–2.5 km deep borehole would likely provide a sufficient temperature gradient to enable the profit-making exploration of geothermal energy, which can then be used for hydrogen production.

An important methodological conclusion of this study is that the low number of stations available to us was sufficient for the satisfactory quality of the calculated seismic model. This opens up the opportunity to obtain interesting results for other areas with seismic small networks that were previously considered too small for seismic tomography inversions.

## Methods

To undertake iterative simultaneous inversions of the velocity model and the source locations, we used LOTOS, passive-source tomography code^28^, which was used for the cases cited above and many other studies. We used the standard version of the LOTOS code, which has been described in detail in previous articles; therefore, here, we will only present the major steps with indications of the key controlling parameters.

The calculations start with determining the first approximations for the source locations using the grid search method and a simplified algorithm to calculate travel times along straight lines. To search for the best source locations, we successively refined the grid (i.e., 4 × 4 × 4 km, 1 × 1 × 1 km, and 0.1 × 0.1 × 0.1 km). We then updated the source determinations using a 3D ray-tracing algorithm based on the bending method^34^.

The 3D distributions of the *P*- and *S*-wave velocity anomalies were parameterized by a set of nodes installed in the study area according to the distribution of the ray paths. We set a minimum grid spacing of 1 km and 0.5 km in the horizontal and vertical directions, respectively. In both cases, the grid spacing is much smaller than the minimum size of anomalies that can be recovered with the existing data. Therefore, the resolution is determined by damping and smoothing, and not by grid spacing. The smaller vertical spacing is defined to make the grid more flexible to describe velocity variations near the surface, where significant relief variations exist. To decrease the effect of the grid geometry to the results, we performed a series of inversions in grids with different basic azimuths (i.e., 0°, 22°, 45°, and 66°) and then averaged the results in a single 3D model with a regularly spaced grid.

For each grid, we performed a simultaneous inversion for the *P*- and *S*-wave velocity anomalies (i.e., d*Vp* and d*Vs*) and the source corrections (i.e., three parameters for the coordinates and one for the origin time). The derived matrix was inverted using the least squares method LSQR algorithm^35,36^. To control the stability of the model, we used amplitude damping (i.e., 0.2 and 0.4 for the *P* and *S* models, respectively) and flattening (i.e., 0.4 and 1.9 for the *P* and *S* models, respectively). The values of these parameters were determined based on the results of the synthetic modeling.

After the inversion step, we updated the 3D velocity model and used it as a new reference model for the next iteration, which started with the relocation of the sources. We then calculated the new matrix and performed the inversion again. In total, we performed five iterations, an optimal compromise between the quality of the solution and the calculation time. In the final step, the average residual deviations in the L1 norm were reduced from 0.077 to 0.070 s for the P-waves and from 0.071 to 0.062 s for the *S*-wave data.

The starting 1D model was defined at several depth levels and linearly interpolated between levels. We used a constant *Vp*/*Vs* ratio of 1.85. The *P*-wave velocities at each level were determined after several runs of the full inversion procedure by adjusting the average velocity according to the results of the previous model. The initial velocity model in the first run was taken from a case of Akutan^11^, where the tomography model was previously constructed for similar geological settings. The values of the *P* velocities in the final 1D reference model are presented in Table 1.

**Table 1:** The reference velocity model used for the inversion of experimental data.

Depth (km)	Vp (km/s)
− 5	3.80
1	4.39
7	5.22
20	5.64
30	7.80

## Data Availability

The full directory with data and program codes to reproduce the results presented in this paper can be downloaded from Zenodo. http://doi.org/10.5281/zenodo.6203017. This compressed file includes a README.pdf file with detailed guidelines on how to perform the calculations.
